# Data‐Driven Scheduling Strategies to Minimize Fatigue in Rotating Night‐Shift Nurses

**DOI:** 10.1155/jonm/9994492

**Published:** 2026-07-20

**Authors:** Su Hyun Kim, Geunsoo Jang, Yeonsu Lee, Sijin Lee, Huiyoung Hwang, Seunghwa Shin, Bomin Jeon, Sol Youn, Hyojung Lee

**Affiliations:** ^1^ College of Nursing, Kyungpook National University, Daegu, South Korea, knu.ac.kr; ^2^ School of Computing and Augmented Intelligence, Arizona State University, Tempe, Arizona, USA, asu.edu; ^3^ Department of Statistics, Kyungpook National University, Daegu, South Korea, knu.ac.kr; ^4^ Department of Nursing, Andong Science College, Andong, South Korea; ^5^ College of Nursing, Suseong University, Daegu, South Korea

**Keywords:** fatigue, models, nurses, shift work schedule, sleep deprivation, sleep hygiene, statistical, workforce management

## Abstract

**Background:**

Shift work is essential for continuous patient care but disrupts circadian rhythms, leading to increased fatigue, burnout, and the risk of errors. Existing guidelines for managing nurse fatigue remain broad and often lack actionable strategies for roster design and staffing.

**Aim:**

This study aimed to identify optimal shift patterns and sleep–wake schedules that minimize fatigue among rotating night‐shift nurses, using mathematical modeling to provide actionable recommendations for nursing management.

**Methods:**

A quantitative observational study was conducted with 273 nurses working 8‐h rotating three‐shift systems in three Korean hospitals. Sleep was monitored over 14 days using wearable devices, supplemented with survey data. Fatigue was estimated using a validated mathematical model based on the two‐process model of sleep regulation, and simulations were applied to evaluate alternative shift sequences and sleep–wake strategies.

**Results:**

Night shifts resulted in the shortest sleep (mean 5.0 h) and the highest fatigue, with over half of work hours predicted to be spent in a fatigued state. Three consecutive day or evening shifts yielded the most favorable outcomes, with cumulative sleep exceeding 26 h and fatigue limited to less than 1 hour. Conversely, three consecutive night shifts resulted in the poorest outcomes, with fatigue affecting 34.7% of work hours. Quick return patterns (< 11 h between shifts) significantly increased fatigue despite comparable sleep durations. Model‐derived recommendations—such as immediate postshift sleep following night duty—improved recovery and reduced cumulative fatigue.

**Conclusions:**

Limiting consecutive night duties, avoiding quick returns, and supporting tailored sleep routines may reduce fatigue among rotating night‐shift nurses.

**Implications for Nursing Management:**

These findings provide managers with practical, evidence‐based guidance for roster design, policy development, and staff education. Incorporating fatigue modeling into workforce planning can enhance nurse well‐being, reduce error risks, and promote sustainable healthcare delivery.

## 1. Introduction

Night‐shift work is an indispensable component of modern healthcare, enabling 24‐h patient care. Globally, approximately 30% of healthcare workers in the European Union and 11.8% in the United States engage in night duty [1]. Shift systems vary across countries, including 12‐h two‐shift systems and 8‐h three‐shift rotations [2]. In the United States, two‐shift systems are more common, and their adoption has been increasing worldwide [3, 4]. However, in Korea, 93.5% of the hospital nurses work under rotating three‐shift systems as of 2019 [5]. Such shift work, involving frequent transitions between day, evening, and night shifts, disrupts circadian rhythms, resulting in poor sleep quality, fatigue, and impaired performance [6].

Fatigue is not only a personal health issue but also a critical managerial concern [7]. It contributes to burnout, diminished job satisfaction, and turnover, while increasing the risk of patient safety incidents [8]. Nearly 92% of shift‐working nurses report fatigue‐related symptoms [8], and one‐third acknowledge fatigue‐related clinical errors within 6 months [9]. These findings underscore the urgent need for evidence‐based scheduling strategies that safeguard staff well‐being and maintain care quality.

Automated nurse rostering systems have been developed and implemented across various countries to improve scheduling efficiency and reduce administrative burden. In the United Kingdom, electronic rostering systems such as Allocate Health Roster are widely used across National Health Service (NHS) trusts [10]. In India, the Rostering and On‐duty Time Automation (ROTA) system has been implemented to automate scheduling processes and reduce manual workload [11]. In Hong Kong, the Hospital Authority introduced an AI‐based rostering system using constraint programming techniques to generate nurse schedules automatically [12]. In addition, these systems primarily focus on operational constraints and staffing requirements and do not explicitly incorporate fatigue, sleep patterns, or circadian rhythms into scheduling decisions.

Previous studies highlight the importance of a multilevel approach to managing sleep‐related fatigue, emphasizing both organizational support for restorative sleep and the promotion of healthy sleep behaviors among nurses [13]. Guidelines for managing nurses’ fatigue recommend optimizing work schedules to account for shift sequences and adequate rest between shifts [14, 15]. However, these guidelines are often overly general and lack the specificity necessary for practical application in clinical settings [16].

Optimizing shift schedules requires strategies that maximize opportunities for restorative sleep while minimizing fatigue [17]. Although previous studies have examined alertness and performance in relation to shift design [18], few have focused on redesigning specific shift sequences and sleep–wake arrangements for nurses working rapidly rotating schedules. Mathematical fatigue models, particularly those based on the two‐process model of sleep regulation [19], offer a promising way to predict fatigue risk and provide nurse managers with practical, data‐driven tools to guide scheduling [20].

This study, therefore, aimed to (1) estimate sleep and fatigue across different shifts among rotating night‐shift nurses and (2) propose optimal work and sleep–wake scheduling strategies. By delivering model‐driven, actionable recommendations, the findings support sustainable staffing policies and provide evidence‐based approaches to enhance nurse well‐being, operational efficiency, and patient safety.

## 2. Methods

### 2.1. Study Design

This study employed a quantitative observational design that combined prospective data collection with computational modeling. Nurses working rotating shifts were observed in real‐world clinical environments without manipulation of their schedules. Sleep–wake data were objectively collected using wearable activity trackers and supplemented with self‐reported surveys capturing demographic and occupational characteristics. A two‐process mathematical model of sleep regulation [19], integrating circadian rhythms and homeostatic sleep pressure, was applied to estimate fatigue trajectories across different shift types. This hybrid design allowed for both empirical assessment of actual sleep behavior and simulation‐based evaluation of work–rest schedules, thereby providing evidence‐based insights into the relationship between rotating shift patterns, sleep duration, and fatigue in hospital nurses.

### 2.2. Participants

Nurses engaged in rotating shift work were recruited from two university hospitals in Daegu and one general hospital in Andong, Republic of Korea, each with more than 1000 beds. Eligibility criteria were (1) nurses currently working at least one night shift per month, (2) aged 19 years or older, (3) ability to complete the study questionnaire, (4) no known allergies to nickel or acrylates, and (5) ownership of a smartphone. Nurses who were pregnant were excluded. This study was approved by the Institutional Review Boards of Kyungpook National University Hospital (KNUH 2021‐11‐004‐001) and Kyungpook National University Chilgok Hospital (KNUCH 2021‐11‐018‐001). Written informed consent was obtained from all participants.

Between January and May 2022, 303 nurses were recruited through hospital network systems. For this analysis, only nurses working within the rotating 8‐h three‐shift system were included, as this was the predominant schedule and differed significantly in sleep patterns from those on 12‐h two‐shift schedules or split schedules. After exclusions (6 due to lack of written consent, 9 working two‐shift schedules, 13 working split schedules, and 2 with incomplete data), the final dataset comprised 273 participants. The shift schedules of the participants were as follows: day shift (7:00 a.m.–3:00 p.m.), evening shift (3:00 p.m.–11:00 p.m.), and night shift (11:00 p.m.–7:00 a.m.).

### 2.3. Data Collection and Measurements

Data were collected over a 14‐day period, beginning with each participant’s first night shift, and included both wearable device monitoring and participant‐reported surveys. Surveys captured sociodemographic and work characteristics. Sleep patterns were continuously monitored using the Fitbit Charge 4 tracker (Fitbit Inc, San Francisco, CA, USA), which recorded data at one‐minute intervals. Key sleep metrics included sleep duration (total time spent asleep), sleep latency (time taken to transition from wakefulness to sleep), awake duration (total time spent awake during designated sleep periods), restless duration (time spent in restlessness, marked by frequent movements), and sleep efficiency (ratio of sleep duration to total time in bed). When multiple sleep episodes occurred after a shift, the longest sleep episode was analyzed.

The Fitbit Charge 4 integrates accelerometry and heart rate variability (HRV) data to track sleep with high precision. Validation studies have demonstrated its accuracy against polysomnography (PSG), reporting a sensitivity of 0.96 for sleep detection, a specificity of 0.61 for wake detection, and an overall accuracy of 0.81 for light sleep detection [21]. Additionally, the device demonstrated a sensitivity of 95% and specificity of 57% for detecting sleep epochs, with minimal discrepancies noted in identifying awakening events and total sleep duration [20].

### 2.4. Statistical and Mathematical Analysis

#### 2.4.1. Prediction of Sleep and Fatigue Durations

Comparisons of sleep and fatigue across shifts were performed using Kruskal–Wallis tests as appropriate. Odds ratios (ORs) were calculated to quantify the likelihood of obtaining adequate sleep (> 7 h) across shifts [22]. Fatigue was estimated using a mathematical model adapted from a previous study [23], which incorporates both homeostatic sleep pressure (accumulated need for sleep) and circadian rhythms. In this study, Fitbit‐generated sleep data, specifically sleep onset time and wake time, were used as input to compute each participant’s sleep pressure. Fatigue duration, defined as periods when sleep pressure exceeded the threshold for sleep but sleep was not possible due to work or environmental demands, was used as a primary outcome. Detailed mathematical equations and parameter specifications used in the modeling are provided in Supporting Table 1.

#### 2.4.2. Optimization of Work and Sleep–Wake Schedules

**FIGURE 1:**
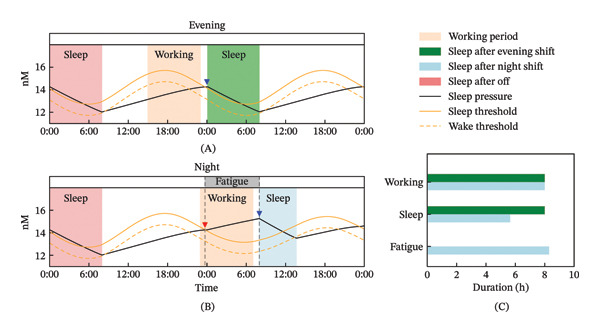
Predicted fatigue periods after evening and night shifts based on the mathematical model. (A) Fatigue patterns following an evening shift. (B) Fatigue patterns following a night shift. (C) Comparison of working hours, sleep duration, and fatigue duration between evening and night shifts. Notes: working periods are shaded orange. Sleep after an evening shift is shaded green. Sleep after a night shift is shaded light blue. Sleep after an off‐day is shaded light coral. Homeostatic sleep pressure is represented by the black line. Sleep threshold is indicated by a solid orange line. Wake threshold is represented by a dashed orange line. Onset of sleep is marked by a blue ∇. The onset of fatigue periods is marked by a red ∇.

Mathematical modeling was applied to evaluate different shift sequences (e.g., day–day–day [DDD], evening–evening–evening [EEE], and night–night–night [NNN]) and to simulate corresponding sleep–wake patterns. For each predefined shift sequence, sleep onset and wake times were systematically varied, and the resulting fatigue duration and total sleep time were calculated using the model. By comparing predicted fatigue and recovery across these arrangements, the study identified schedules that minimized fatigue while maximizing recovery opportunities. The modeling framework, based on the two‐process model of sleep regulation [19] and illustrated in Figure 1, demonstrates how night shifts (11:00 p.m.–7:00 a.m.) elevate homeostatic sleep pressure, leading to extended fatigue periods. By analyzing the effects of shift sequence and sleep–wake schedules on sleep and fatigue duration, the model aims to identify optimal scheduling strategies that reduce fatigue while supporting adequate recovery between shifts.

## 3. Results

### 3.1. Participants’ Characteristics

The demographic and occupational characteristics of the 273 participants are summarized in Table 1. The majority of the participants were in their 20s (82%), female (94.1%), and living without children (95.6%). Additionally, 66.3% were employed in general wards and 50.9% had 3–10 years of clinical experience.

**TABLE 1:** Participants’ characteristics (*N* = 273).

Variables	*N* (%)
Age	
20–29 years	224 (82.1%)
30–39 years	43 (15.8%)
≥ 40 years	6 (2.2%)
Sex, female	257 (94.1%)
Married, single	238 (87.2%)
Living situation	
Living alone	46 (16.8)
Living with family (without children)	215 (78.8)
Living with children	12 (4.4)
Work units	
General ward	181 (66.3%)
Intensive care unit	63 (23.1%)
Emergency room	24 (8.8%)
Operating room	5 (1.8%)
Clinical career (years)	
≤ 1	29 (10.6%)
> 1 and ≤ 3	82 (30%)
> 3 and ≤ 10	139 (50.9%)
> 10	23 (8.5%)

### 3.2. Prediction of Sleep and Fatigue Durations Across Shifts

During the study period, a total of 1321 day shifts, 766 evening shifts, and 753 night shifts were observed (Table 2). The Kruskal–Wallis test revealed significant differences in both sleep duration (*χ* ^2^ = 516.76, *p* < 0.001) and sleep latency (*χ* ^2^ = 175.82, *p* < 0.001) across work shifts over a 14‐day period. Post hoc analyses indicated that these differences were significant across most comparisons, except between day shifts and off‐shifts. Specifically, sleep duration was longest following evening shifts (7.2 ± 2.2 h), with a mean sleep latency of 32.7 ± 39.4 min. In contrast, sleep duration was shortest after night shifts (5.0 ± 2.2 h), with a sleep latency of 21.9 ± 20.4 min. OR analysis further quantified these differences. The night shift was associated with a substantially lower likelihood of adequate sleep (> 7 h) compared with the evening shift (OR = 0.09 and 95% CI: 0.07–0.12) and the day shift (OR = 0.21 and 95% CI: 0.16–0.26). Additionally, the day shift was associated with a lower likelihood of adequate sleep compared with the evening shift (OR = 0.45 and 95% CI: 0.36–0.57).

**TABLE 2:** Sleep duration, sleep latency, and fatigue proportion, total number of shifts during work across shifts.

Shift type	Total number of shifts	Sleep duration (hours)^a^ (Mean ± SD)	Sleep latency (minutes)^a^ (Mean ± SD)	Fatigue proportion during work (%)^b^
Day	1321 (37.5%)	5.4 ± 2.5	22.7 ± 25.0	36.29%
Evening	766 (21.7%)	7.2 ± 2.2	32.7 ± 39.4	2.58%
Night	753 (21.4%)	5.0 ± 2.2	21.9 ± 20.4	51.42%
Off	685 (19.4%)	5.9 ± 2.4	26.6 ± 31.6	—

^a^Sleep duration (hours) and sleep latency (minutes) are observed sleep statistics calculated from Fitbit sleep data.

^b^Fatigue proportion during work (%) is a model‐derived estimate calculated using the mathematical model.

Model‐predicted fatigue patterns are presented in Figure 1. Evening shifts (Figure 1A) did not induce fatigue, whereas night shifts (Figure 1B) were associated with prolonged fatigue periods. Figure 1C illustrates that sleep duration following night shifts decreased to less than 6 hours, whereas fatigue duration extended to approximately 8 hours. The proportion of work time spent in a fatigued state was highest during the night shift (51.42%) and lowest during the evening shift (2.58%). These findings underscore the significant impact of night shifts on sleep deprivation and fatigue accumulation.

### 3.3. Shift Schedule Arrangements to Enhance Sleep

Among the observed schedules, three consecutive workdays were most common (29.9%), followed by two consecutive workdays (29.0%), four consecutive workdays (20.9%), one workday (11.0%), five consecutive workdays (7.2%), and six consecutive workdays (1.48%). Given that three consecutive workdays represent the most frequently observed pattern, this study focuses on 3‐day work sequences as a primary unit for subsequent analysis. This choice is also consistent with previous studies that have used three consecutive workdays to capture cumulative fatigue effects [3, 24].

Model evaluations of common 3‐day schedules showed that the DDD sequence yielded the highest cumulative sleep (26.55 h) and the lowest fatigue duration (0.37 h) over three consecutive workdays, as summarized in Table 3. The EEE sequence produced similar outcomes, with high cumulative sleep duration (26.12 h) and low fatigue duration (0.70 h). In contrast, sequences involving night shifts were associated with considerably higher fatigue, exceeding 10% in all sequences. The NNN schedule was the least favorable, with fatigue affecting 34.74% of work time.

**TABLE 3:** Predictions from the mathematical model for sleep and fatigue across three consecutive shifts.

Shift schedule	Cumulative sleep duration (hours)	Cumulative fatigue duration (hours)	Fatigue proportion (%)
DDD	26.55	0.37	0.52
DDE	26.51	1.55	2.15
DDN	21.42	9.32	12.94
DED	26.27	1.60	2.22
DEE	26.46	2.98	4.14
DEN	23.35	10.73	14.91
DNN	15.11	19.35	26.88
EDD	24.58	7.49	10.40
EDE	25.35	4.10	5.69
EDN	22.48	11.65	16.19
EED	25.38	3.47	4.81
EEE	26.12	0.70	0.98
EEN	23.27	8.25	11.46
ENN	20.93	17.00	23.61
NNN	19.18	25.01	34.74

*Note:* D = day shift (7:00 a.m.–3:00 p.m.), E = evening shift (3:00 p.m.–11:00 p.m.), N = night shift (11:00 p.m.–7:00 a.m.).

Additionally, hybrid shift schedules that included evening–day transitions (e.g., evening–day–day [EDD], evening–day–evening [EDE], and evening–evening–day [EED]) provided cumulative sleep duration comparable to the DDD schedule (24.58, 25.35, and 25.38 h, respectively). However, these schedules resulted in considerably higher fatigue duration (7.49, 4.10, and 3.47 h, respectively).

### 3.4. Sleep–Wake Scheduling to Reduce Fatigue Duration

In this section, we examine sleep–wake schedules suggested by the mathematical model for different shift sequences. As in the previous section, we focus on 3‐day work sequences. Prior to the start of each sequence, an off‐shift is assumed, during which sufficient sleep is obtained, allowing circadian rhythms to stabilize and fatigue levels to remain low.

Table 4 and Supporting Figure 1 present sleep–wake schedules designed to reduce fatigue. For example, under the DDD schedule, a suggested sleep period following the second day shift was 9:45 p.m.–6:00 a.m. Overall, sleeping between 9:00 p.m. and 10:00 p.m. during consecutive day shifts appears to be an effective schedule for minimizing fatigue duration.

**TABLE 4:** Suggested sleep–wake schedules for three consecutive shifts based on the mathematical model.

Shift schedule	Day 1 sleep period	Day 2 sleep period	Day 3 sleep period
DDD	09:45 p.m.–06:00 a.m.	09:45 p.m.–06:00 a.m.	09:30 p.m.–07:30 a.m.
DDE	09:45 p.m.–06:00 a.m.	09:45 p.m.–07:15 a.m.	12:00 a.m.–08:15 a.m.
DDN	09:45 p.m.–06:00 a.m.	09:45 p.m.–07:15 a.m.	08:00 a.m.–01:15 p.m.
DED	09:45 p.m.–07:15 a.m.	12:00 a.m.–06:00 a.m.	09:00 p.m.–07:30 a.m.
DEE	09:45 p.m.–07:15 a.m.	12:00 a.m.–08:15 a.m.	12:00 a.m.–08:15 a.m.
DEN	09:45 p.m.–07:15 a.m.	12:00 a.m.–08:15 a.m.	08:00 a.m.–01:15 p.m.
DNN	09:45 p.m.–07:15 a.m.	08:00 a.m.–01:00 p.m.	08:00 a.m.–01:45 p.m.
EDD	12:00 a.m.–08:00 a.m.	12:15 a.m.–08:00 a.m.	12:00 a.m.–11:00 a.m.
EDE	12:00 a.m.–08:00 a.m.	12:15 a.m.–10:45 a.m.	02:15 a.m.–11:00 a.m.
EDN	12:00 a.m.–08:00 a.m.	12:15 a.m.–10:45 a.m.	10:00 a.m.–03:45 p.m.
EED	12:00 a.m.–10:45 a.m.	02:15 a.m.–08:00 a.m.	12:00 a.m.–11:00 a.m.
EEE	12:00 a.m.–10:45 a.m.	02:15 a.m.–10:45 a.m.	02:15 a.m.–11:00 a.m.
EEN	12:00 a.m.–10:45 a.m.	02:15 a.m.–10:45 a.m.	10:00 a.m.–03:45 p.m.
ENN	12:00 a.m.–10:45 a.m.	10:00 a.m.–03:30 p.m.	10:00 a.m.–04:15 p.m.
NNN	10:00 a.m.–04:00 p.m.	10:00 a.m.–04:30 p.m.	10:00 a.m.–04:30 p.m.

*Note:* D = day shift (7:00 a.m.–3:00 p.m.), E = evening shift (3:00 p.m.–11:00 p.m.), N = night shift (11:00 p.m.–7:00 a.m.).

For night shifts, immediate sleep after completing a shift, lasting between 5 and 6.5 h, substantially mitigated fatigue. For instance, under the day–night–night (DNN) schedule, a suggested sleep–wake schedule on Day 2 was from 8:00 a.m. (immediately after the night shift) to 1:00 p.m. Similarly, under the EED schedule, sleeping from 2:15 a.m. to 8:00 a.m. after the second evening shift was effective.

**FIGURE 2:**
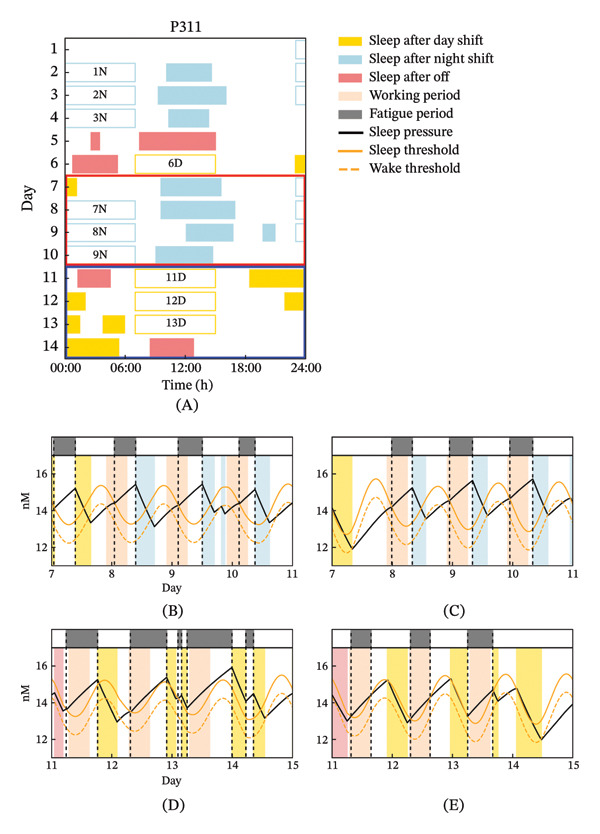
Analysis of a participant’s work schedule and sleep pattern with short sleep duration. (A) Work schedule and sleep pattern over Days 1–14. (B) Actual sleep patterns during night shifts (Days 7–11). (C) Recommended sleep patterns based on the mathematical model for night shifts (Days 7–11). (D) Actual sleep patterns during day shifts (Days 11–14). (E) Recommended sleep patterns based on the mathematical model for day shifts (Days 11–14). *Notes*: Sleep after a day shift is represented by the yellow bar. Nap before a night shift is represented by the blue bar. Sleep after a night shift is represented by the sky blue bar. Sleep after an off‐day is represented by the light coral bar. Homeostatic sleep pressure is represented by the black line. Sleep threshold is indicated by a solid orange line. Wake threshold is represented by a dashed orange line. Fatigue periods are shown as gray bars.

Figure 2 illustrates a comparison between the actual and modeled sleep–wake schedules for a participant with short sleep duration (≤ 6.31 h), providing an example of how these suggested schedules may be applied. During the NNN schedule (Days 7–10), following the suggested sleep–wake schedule improved both sleep duration and fatigue outcomes. The modeled sleep–wake schedule (Figure 2C) demonstrated superior outcomes compared with the participant’s actual sleep (Figure 2B). A particularly pronounced example occurred during the DDD schedule (Days 11–14), where the participant’s actual sleep on Day 12 was limited to just 3.75 h (Figure 2D). By contrast, the suggested sleep–wake schedule of sleeping from 10:00 p.m. to 6:00 a.m. may have allowed for approximately 8 hours of sleep (Figure 2E). Over the 8‐day period from Days 7 to 14, the participant’s actual sleep totaled 50 h with fatigue accumulation of 64.25 h. In comparison, adherence to the modeled sleep–wake schedule increased total sleep to 59.5 h and reduced fatigue to 51.5 h.

## 4. Discussion

This study identified shift patterns and sleep–wake schedules associated with reduced fatigue using mathematical modeling, offering evidence‐based insights to guide fatigue management strategies for shift‐working nurses. The findings underscore the importance of optimizing both work schedules and sleep–wake routines to mitigate the adverse effects of fatigue in rotating night‐shift nurses.

Night shifts, which are inherently misaligned with circadian rhythms, were found to significantly reduce sleep duration and prolong fatigue, consistent with previous studies [15, 25]. In this study, participants’ actual sleep following night shifts was typically less than 6 h, reflecting the difficulty of maintaining sleep after night shifts due to rising circadian wakefulness during daylight hours. Insufficient sleep prevents the dissipation of accumulated sleep pressure, leading to persistent fatigue upon waking. The highest proportion of fatigue (51.42%) was observed during night shifts, indicating an increased risk to patient safety if countermeasures are not implemented. Day shifts also showed increased fatigue (36.29%), attributed to early start times coinciding with the “window of circadian low” [26]. These findings reinforce the need to align work schedules with circadian physiology to reduce fatigue [27].

When evaluating specific shift arrangements, the DDD and EEE schedules were identified as the most favorable for minimizing fatigue and maximizing cumulative sleep duration. In contrast, the NNN schedule exhibited the highest fatigue levels and the shortest sleep durations, supporting previous evidence that more than three consecutive night shifts compromise sleep quality [28]. Shift patterns involving “quick returns”—such as EDD, evening–day–evening (EDE), and EED—provided similar sleep durations to the DDD schedule but substantially higher fatigue levels. This finding aligns with research showing that reduced recovery of less than 11 h between shifts markedly increases fatigue [15]. These results provide actionable insights into how shift sequences impact fatigue, emphasizing the importance of deliberate scheduling to mitigate its effects [25]. For nurse managers, these results provide clear scheduling guidance that better aligns with nurses’ physiological needs, such as avoiding three consecutive night shifts, limiting or eliminating quick return patterns, and prioritizing consecutive day or evening shifts to balance recovery and service coverage.

Previous studies [23, 29] focused on individual sleep behavior and alertness using wearable data and mathematical modeling, whereas our study extends this approach by incorporating shift schedules to evaluate and optimize fatigue, providing a framework for fatigue‐aware scheduling. The model‐derived sleep–wake schedules suggest that relatively simple adjustments to sleep timing can mitigate fatigue. For example, adopting early bedtimes during day‐shift sequences (9:00 or 10:00 p.m.–6:00 a.m.) and immediate sleep after night duty (8:00 or 10:00 a.m.–1:15 or 3:45 p.m.) improved recovery. Such strategies can be integrated into staff education, orientation, and occupational health programs, helping nurses align personal routines with organizational demands. Implementing these practices may serve as effective interventions to support both nurse well‐being and care quality.

The findings of this study provide nurse managers with clear, evidence‐based strategies to support fatigue management in shift‐working nurses. First, careful roster design is essential: scheduling more than three consecutive night shifts should be avoided, quick returns of less than 11 h between shifts should be minimized or eliminated, and sequences of consecutive day or evening shifts should be prioritized to allow sufficient recovery. In addition, incorporating model‐derived sleep–wake recommendations into staff education can encourage healthy sleep practices, such as going to bed earlier during consecutive day shifts and taking immediate postshift sleep following night duty.

From a workforce planning perspective, predictive fatigue modeling can serve as a practical scheduling tool to anticipate periods of high risk, balance staffing needs, and support advocacy for safer rostering practices. At the policy level, collaboration with institutional leaders and regulators is necessary to establish minimum recovery times and limits on consecutive night duties as part of staffing standards.

This study has several limitations. First, the use of wearable devices and self‐reported data introduces potential measurement errors and reporting biases. Second, the study was conducted in Korean hospitals with rapidly rotating three‐shift systems, which may limit generalizability to other settings. Third, real‐world implementation may be constrained by staffing shortages, workload demands, and contractual agreements. Despite these limitations, our approach provides useful insights and can be adapted to a variety of scheduling systems, including two‐shift arrangements commonly used in other countries. The modeling approach can be straightforwardly applied to evaluate and optimize fatigue in those contexts. Future research should investigate the long‐term outcomes of optimized scheduling on sleep health, nurse retention, job satisfaction, and patient safety across diverse healthcare settings.

## 5. Conclusion

By combining wearable data and mathematical modeling, this study demonstrates that deliberate scheduling and tailored sleep–wake schedules can substantially reduce fatigue among rotating shift nurses. For nurse managers, the findings provide actionable strategies for roster design, staff education, and workforce planning. Addressing fatigue through evidence‐based scheduling is an essential step toward promoting nurse well‐being, ensuring patient safety, and sustaining the healthcare workforce.

## Author Contributions

Study design: Su Hyun Kim and Hyojung Lee; data collection: Su Hyun Kim, Seunghwa Shin, Bomin Jeon, and Sol Youn; data analysis: Hyojung Lee, Geunsoo Jang, Huiyoung Hwang, Yeonsu Lee, and Sijin Lee; manuscript writing: Su Hyun Kim, Hyojung Lee, Geunsoo Jang, Huiyoung Hwang, Yeonsu Lee, and Sijin Lee.

## Funding

This research was supported by the Basic Science Research Program through the National Research Foundation of Korea, funded by the Ministry of Science, ICT, and Future Planning (NRF‐2020R1A2C110161015) (PI: Su Hyun Kim) and the National Research Foundation of Korea (NRF) grant, funded by the Korean government (RS‐2022‐NR070839, RS‐2022‐NR071821) (PI: H. Lee).

## Ethics Statement

This study was conducted in accordance with the principles of the Declaration of Helsinki. It was approved by the Institutional Review Boards of Kyungpook National University Hospital (KNUH 2021‐11‐004‐001) and Kyungpook National University Chilgok Hospital (KNUCH 2021‐11‐018‐001).

## Conflicts of Interest

The authors declare no conflicts of interest.

## Supporting Information

Additional supporting information can be found online in the Supporting Information section.

## Supporting information


**Supporting Information** Supporting Information provides additional methodological details and figures related to the mathematical fatigue model and recommended sleep–wake patterns. Supporting Table 1 provides a detailed description of the mathematical fatigue model, including the formulation of homeostatic sleep pressure, circadian rhythm, sleep and wake thresholds, and baseline assumptions used for model validation. Supporting Figure 1 illustrates recommended sleep–wake patterns for three consecutive work shifts under various shift sequences (day, evening, and night shifts), derived from the mathematical fatigue model.

## Data Availability

The data that support the findings of this study are available on request from the corresponding author. The data are not publicly available due to privacy or ethical restrictions.
