# Supplementary material for: Data‐Driven Scheduling Strategies to Minimize Fatigue in Rotating Night‐Shift Nurses
Source: J Nurs Manag. 2026 Jul 20;2026:9994492. doi: 10.1155/jonm/9994492 (PMC13385972; doi:10.1155/jonm/9994492)
Supplement: Supplementary file 1 — Supporting Information Supporting Information provides additional methodological details and figures related to the mathematical fatigue model and recommended sleep–wake patterns. Supporting Table 1 provides a detailed description of the mathematical fatigue model, including the formulation of homeostatic sleep pressure, circadian rhythm, sleep and wake thresholds, and baseline assumptions used for model validation. Supporting Figure 1 illustrates recommended sleep–wake patterns for three consecutive work shifts under various shift sequences (day, evening, and night shifts), derived from the mathematical fatigue model. [file JONM-2026-9994492-s001.docx]

**Supplementary Materials**

**Supplementary Table 1.** Mathematical Model of Fatigue

| **Indicators** | **Description** |
| --- | --- |
| Homeostatic Sleep Pressure (*H(t)*) | The homeostatic sleep pressure *H(t)* increases during wakefulness and dissipates during sleep. It was derived using participants’ continuously monitored sleep-wake patterns:  *H(t)* = *f*(sleep onset, wake times)  where accumulation reflects hours awake, and dissipation reflects hours asleep. |
| Circadian Rhythm (*C(t)*) | Circadian rhythm *C(t)* was modeled as a sinusoidal function modulated by light exposure:  *C(t) = g(I(t))*  where *I(t)* is light exposure at time t.  • Baseline assumptions:  o 0 lux during sleep  o 250 lux during wake periods |
| Sleep and Wake Threshold ($H^{+}\left( t \right)$ and $H^{-}\left( t \right)$) | The model defines thresholds at which transitions between wakefulness and sleep occur:  $H^{+}\left( t \right)=\frac{2.46-A+C\left( t \right)}{v_{vh}}$  $H^{-}\left( t \right)=\frac{1.45-A+C\left( t \right)}{v_{vh}}$  where   - A=10.2 - $v_{vh}=1.01$ - Sleep threshold $H^{+}\left( t \right)$: when exceeded, the individual is likely to fall asleep. - Wake threshold $H^{-}\left( t \right)$: when dropped below, the individual is likely to wake up. |
| Fatigue periods |  Fatigue period: when $H(t)>H^{+}\left( t \right)$ but the participant cannot sleep due to environmental/occupational demands.   Fatigue duration: cumulative hours spent in fatigue periods. |
| Baseline assumptions for validation |  Without external influences, participants were assumed to follow a regular schedule (12:00 a.m. – 8:00 a.m. sleep).   These assumptions allowed comparative evaluation across shift sequences. |


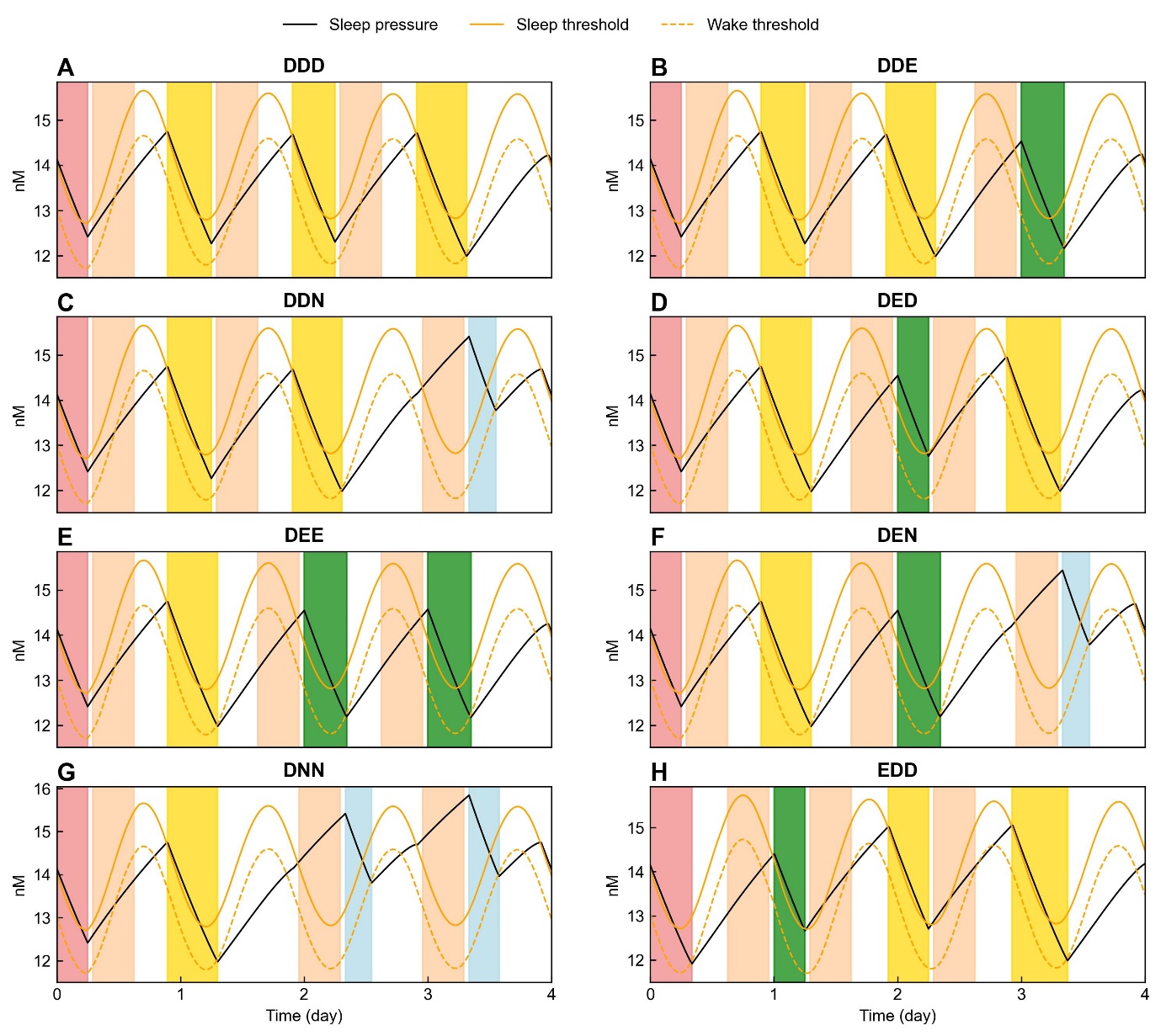


**Supplementary Figure 1.** Recommended sleep–wake patterns for three consecutive work shifts, derived from the mathematical fatigue model.

**A.** DDD schedule **B.** DDE schedule **C.** DDN schedule **D.** DED schedule **E.** DEE schedule **F.** DEN schedule **G.** DNN schedule **H.** EDD schedule.

Notes: Optimal sleep windows are indicated for each shift type, demonstrating strategies to reduce cumulative fatigue and enhance recovery across consecutive workdays.

D = Day shift (7:00 a.m. – 3:00 p.m.), E = Evening shift (3:00 p.m. – 11:00 p.m.), N = Night shift (11:00 p.m. – 7:00 a.m.).

Homeostatic sleep pressure is represented by the black line. Sleep threshold is indicated by a solid orange line. Wake threshold is represented by a dashed orange line.


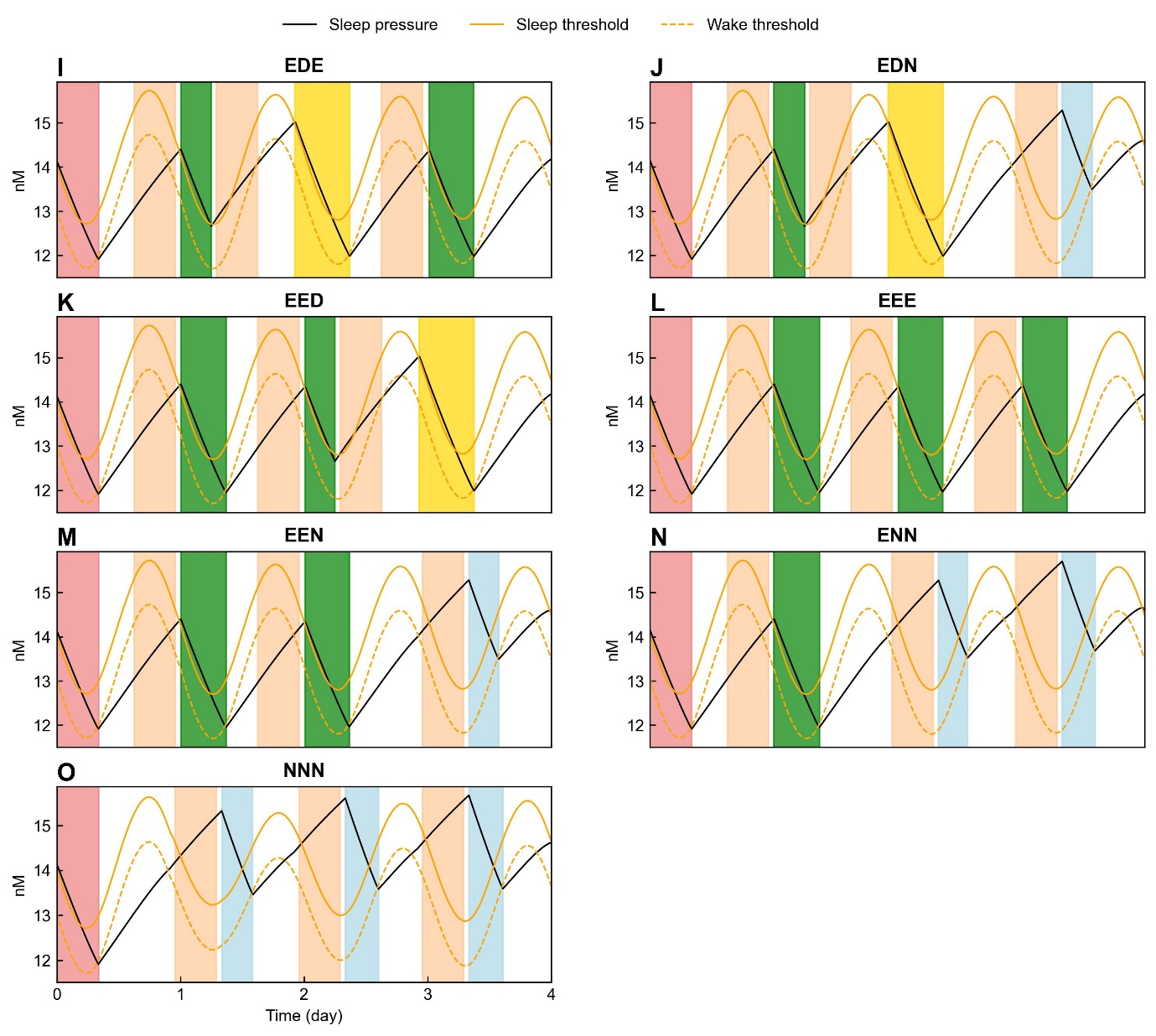


**Supplementary Figure 1 (continued).** Recommended sleep–wake patterns for three consecutive work shifts, derived from the mathematical fatigue model (continued).

**I.** EDE schedule **J.** EDN schedule **K.** EED schedule **L.** EEE schedule **M.** EEN schedule **N.** ENN schedule **O.** NNN schedule.

Notes: Optimal sleep windows are indicated for each shift type, demonstrating strategies to reduce cumulative fatigue and enhance recovery across consecutive workdays.

D = Day shift (7:00 a.m. – 3:00 p.m.), E = Evening shift (3:00 p.m. – 11:00 p.m.), N = Night shift (11:00 p.m. – 7:00 a.m.).

Homeostatic sleep pressure is represented by the black line. Sleep threshold is indicated by a solid orange line. Wake threshold is represented by a dashed orange line.
